# OLDER ADULTS’ HEALTH AND SOCIAL ENGAGEMENT: THE MEDIATING ROLE OF SUBJECTIVE LIFE EXPECTANCY

**DOI:** 10.1093/geroni/igz038.2555

**Published:** 2019-11-08

**Authors:** Dahee Kim, Kyuho Lee

**Affiliations:** 1 Iowa State University, Ames, Iowa, United States; 2 Daegu University, Gyeongsan, Republic of Korea

## Abstract

Older adults’ mental and physical health is likely to limit social engagement, but their perception of how much time they have left, according to the socio-emotional selectivity theory, might influence it as well. The aim of the research is to investigate the mediating effect of subjective life expectancy (SLE) on the pathways from older adults’ mental health and functional limitation to volunteering and contacts with close relationships. The current research used data of 5,285 older adults aged 50 to 75 from the Health and Retirement Study collected in 2014. Structural equation modeling was performed to investigate the direct effect of older adults’ depressive symptoms and functional limitation on volunteering and contact with close relationships. Predictors’ indirect effects via SLE was also assessed. The results indicated that older adults’ higher depressive symptoms and functional limitations significantly decreased volunteering time and frequency of contacts with close relationships. Older adults’ SLE attenuated the effects of depressive symptoms and functional limitations on their volunteering time and frequency of contact with close relationships. The findings describe the mechanism of how older adults engage in volunteering and contact with close relationships through their perception of remaining time. Further, this research highlights SLE as a motivator for encouragement of older adults’ social engagement.

